# Silica nanoparticles for the layer-by-layer assembly of fully electro-active cytochrome *c *multilayers

**DOI:** 10.1186/1477-3155-9-59

**Published:** 2011-12-30

**Authors:** Sven C Feifel, Fred Lisdat

**Affiliations:** 1 Biosystems Technology, University of Applied Sciences, Wildau 15745, Germany

## Abstract

**Background:**

For bioanalytical systems sensitivity and biomolecule activity are critical issues. The immobilization of proteins into multilayer systems by the layer-by-layer deposition has become one of the favorite methods with this respect. Moreover, the combination of nanoparticles with biomolecules on electrodes is a matter of particular interest since several examples with high activities and direct electron transfer have been found. Our study describes the investigation on silica nanoparticles and the redox protein cytochrome *c *for the construction of electro-active multilayer architectures, and the electron transfer within such systems. The novelty of this work is the construction of such artificial architectures with a non-conducting building block. Furthermore a detailed study of the size influence of silica nanoparticles is performed with regard to formation and electrochemical behavior of these systems.

**Results:**

We report on interprotein electron transfer (IET) reaction cascades of cytochrome *c *(cyt *c*) immobilized by the use of modified silica nanoparticles (SiNPs) to act as an artificial matrix. The layer-by-layer deposition technique has been used for the formation of silica particles/cytochrome *c *multilayer assemblies on electrodes. The silica particles are characterized by dynamic light scattering (DLS), Fourier transformed infrared spectroscopy (FT-IR), Zeta-potential and transmission electron microscopy (TEM). The modified particles have been studied with respect to act as an artificial network for cytochrome *c *and to allow efficient interprotein electron transfer reactions. We demonstrate that it is possible to form electro-active assemblies with these non-conducting particles. The electrochemical response is increasing linearly with the number of layers deposited, reaching a cyt *c *surface concentration of about 80 pmol/cm^2 ^with a 5 layer architecture. The interprotein electron transfer through the layer system and the influence of particle size are discussed.

**Conclusions:**

This study demonstrates the ability to construct fully electro-active cyt *c *multilayer assemblies by using carboxy-modified silica nanoparticles. Thus it can be shown that functional, artificial systems can be build up following natural examples of protein arrangements. The absence of any conductive properties in the second building block clearly demonstrates that mechanisms for electron transfer through such protein multilayer assemblies is based on interprotein electron exchange, rather than on wiring of the protein to the electrode.

The construction strategy of this multilayer system provides a new controllable route to immobilize proteins in multiple layers featuring direct electrochemistry without mediating shuttle molecules and controlling the electro-active amount by the number of deposition steps.

## Background

Silica particles at the nano- and microscale are widely used in various areas of science [1,2]. Their universalism is due to the ease of preparation and possibility of controlling size, a high surface-to-volume ratio, and the biocompatibility of silica. Accordingly, several synthetic approaches for the synthesis of silica nanoparticles (SiNPs) are available [3-5]. Different surface modification protocols have been developed in order to immobilize various biomolecules such as enzymes, proteins, and DNA [6-8]. As a stable solid support for such molecules or biomolecular conjugates they have opened the door to applications in sensors [9], drug delivery system [10], and smart materials [11]. For bioanalytical systems sensitivity and biomolecule activity are very critical issues. SiNPs can provide an artificial matrix and thus keeping and enhancing the bioactivity. Various approaches have been developed for increasing the sensitivity of sensors [12-18]. Particular advantage is the defined increase in surface concentrations of the recognition element. The immobilization of proteins into multilayer systems by the layer-by-layer deposition has become one of the favorite methods [19-24]. Moreover, the combination of nanoparticles with biomolecules on electrodes is a matter of particular interest since several examples with direct electron transfer have been found [25-29]. By the use of the redox protein cyt *c *and the polyelectrolyte polyaniline sulfonic acid (PASA) fully electro-active multilayers have been constructed on electrodes. Raising the number of layers a continuous increase of the voltammetric cyt *c *signal has been achieved with this system and can be used for the detection of superoxide radicals with enhanced sensitivity [30,31]. Such multilayer assemblies have recently been shown to enable incorporation of enzymes and establish communication to the electrode, thus allowing the construction of different analytical signal chains [24,32,33]. SiNPs have already been used in biosensors since the high surface area of nano-sized silica particles can increase the surface molecule loading and thus lead to a higher performance of the biosensor [25,34-36].

In this study we want to show that silica nanoparticles can be used as building blocks in layered architecture of proteins on electrodes. It can be expected that the particle size and the surface charge of the used silica nanoparticles play a key role in modulating the properties of such multilayer architectures.

We propose that multilayer assemblies with the redox protein cyt *c *and different-sized surface modified SiO_2 _particles, ranging from 5 - 60 nm can lead to novel architectures in which the cyt *c *molecules can exhibit direct electrochemistry. Thereby we combine the layer-by-layer technique and modified SiNPs for construction of fully electro-active cyt *c *multilayer electrodes. The conditions of assembly formation and the stability are determined by quarz crystal microbalance (QCM). The electrochemical properties of the multilayer architectures are analyzed by cyclic voltammetry (CV). Special focus is on the size influence of the particles and the mediator-free electron transfer ability of the multilayer assembly.

## Results and Discussion

We select cytochrome *c *and silica nanoparticles for this study on the basis of the following considerations. First, silica nanoparticles can be synthesized and functionalized in a variety of sizes, and characterized by a narrow particle size distribution. Second, silica is a dielectric material, which does not absorb light or conduct electrons. As an inert host, it may enable the assembly of biological materials (e.g. cyt *c*) while keeping its natural function.

Finally, the pI values of carboxy-modified silica colloids and cytochrome *c *are acidic and basic respectively [37,38], and therefore, sufficient Coulomb interaction can be expected between silica and the opposite charged cytochrome *c*.

### Synthesis and characterization of SiNPs

**Figure 1:**
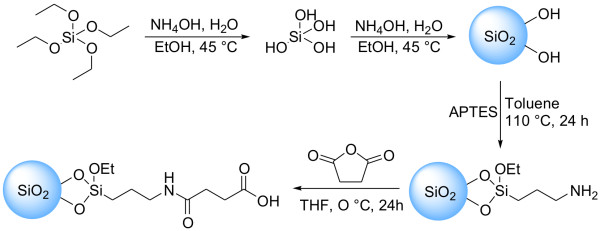
**Scheme SiNP synthesis**. Synthetic route for the synthesis of SiNPs and the surface modification by self-assembly of APTES followed by the coupling reaction of succinic acid anhydride for the introduction of the carboxylic group onto the SiNPs surface.

**Figure 2:**
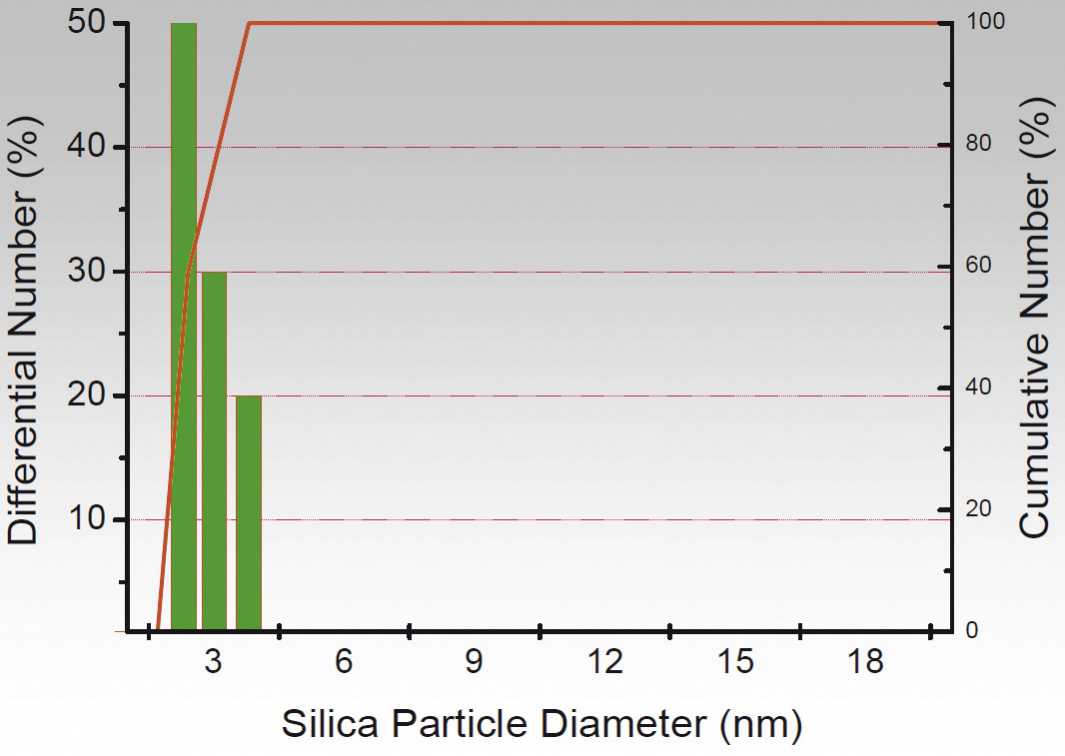
**Dynamic light scattering measurement**. Dynamic light scattering (DLS) measurement of the synthesized silica nanoparticles. For example the unmodified bare silica spheres (Ø = 2.5 nm in average, PI = 0.07).

**Figure 3:**
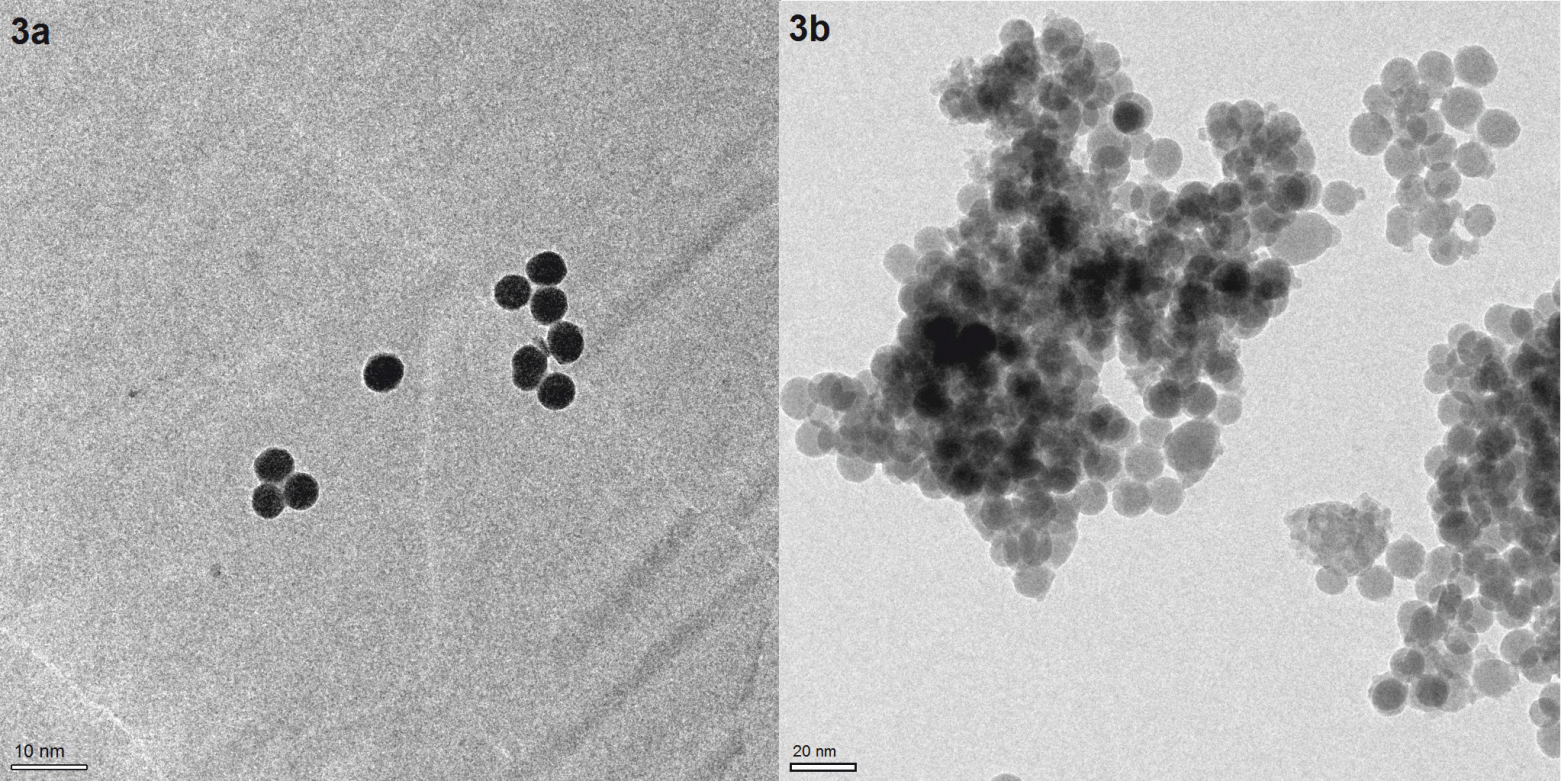
**TEM**. TEM image of synthesized silica nanoparticles. (a) 5 nm silica particles, (b) 10 nm silica particles (200 kV operation voltage, resolution 0.24 nm).

Silica nanoparticles are prepared by the Stöber method. In order to enhance surface charge of the SiO_2 _spheres, they are modified by grafting a γ-aminopropyl silane (APTES) onto the silica particles, followed by nucleophilic addition reaction using succinic acid anhydride and the amino function of the grafted silane to finally obtain the carboxy-modified SiNPs. Figure 1 illustrates the major steps of the synthetic route. The surface-modified SiO_2 _particles are first characterized in terms of size and polydispersity using dynamic light scattering (DLS) - see Figure 2. The average diameter of the different batch sized particles are found to be around 5, 8, 10, 15, 20, 40 and 60 nm, respectively, with a polydispersity index (PI) between 0.05 - 0.15 which implies that they are of fairly uniform size. These results are confirmed by TEM data (Figure 3) which show the SiNPs not to be aggregated. The size as estimated from the TEM data is slightly smaller than the size found by DLS because in DLS the hydrodynamic radius of the particles is measured.

**Figure 4:**
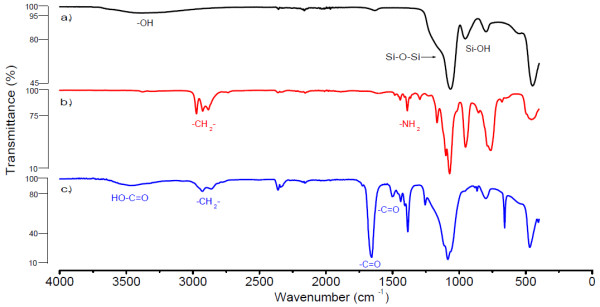
**FT-IR**. FT-IR spectra of synthesized and modified silica nanoparticles. (**a**) SiO_2_, (**b**) SiO_2_-NH_2_, (**c**) SiO_2_-CO_2_H. ATR (Diamond) is used with a resolution 8 cm^-1^, scans 16, in the range of 400-4,000 cm^-1^.

Since the Zeta-potential is an important factor for controlling the surface charge of the SiNPs suspensions further characterization has been performed with this method. Table 1 shows the Zeta-potentials of the unmodified and modified SiO_2 _spheres. It is obvious that the Zeta-potentials of SiO_2 _spheres correspond to the introduced charge on their surface. FT-IR analysis is further used to confirm the introduction of the different functional groups onto the surface of SiO_2 _spheres. Figure 4a shows the FT-IR spectrum of the bare SiO_2 _spheres. The absorption peak at 1020 - 1110 cm^-1 ^is assigned to the Si-O-Si asymmetric stretching vibration, and the peaks at 960 cm^-1 ^are ascribed to the asymmetric bending and stretching vibration of Si-OH, respectively. By contrast, Figure 4b shows the asymmetrical deformation vibration of the amino group at 1425 and 900 cm^-1^, indicating the amino groups were fixed onto the SiO_2 _particle surface successfully. The further modification of the SiO_2_-NH_2 _particles with succinic acid anhydride for the introduction of the carboxyl function can be seen in the FT-IR spectra through the occurrence of the carboxylate peak, and the peaks at 1725, 1461 cm^-1^, all correspond to the vibration peaks of the -COOH (Figure 4c). Thus, it can be concluded that a sequential modification on the SiO_2 _spheres surface takes place.

**Table 1:** Zeta-potentials.

SiO_2_-nanoparticles	Bare SiO_2 _spheres	Modified SiO_2_-NH_2 _spheres	Modified SiO_2_-CO_2_H spheres
Zeta-potential (mV)	-41.44	+39.63	-54.71

Zeta-potentials of the synthesized and modified SiNPs

### Adsorption characteristics of SiNPs and cyt *c*

**Figure 5:**
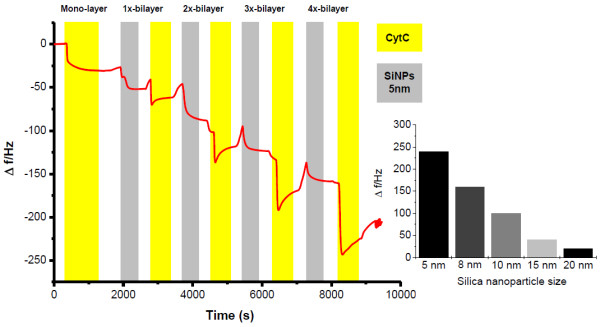
**QCM - Multilayer assembly**. Multilayer formation of a 4-bilayer assembly of cyt *c*/SiNP Ø = 5 nm). The adsorption behavior of SiNPs (Ø = 5 nm) to cytochrome *c *and *vice versa *is shown for a 4-bilayer architecture. QCM chips modified with self-assembled monolayer of MUA/MU are successively flushed with solutions of SiNPs (0.5 mg/mL) and cyt *c *(20 μM), flow rate 25 μL/min. Inset: QCM - Frequency change during the formation of 4-bilayer (cyt *c*/SiNPs) assemblies on a QCM chip by the use of different sized SiNPs (5, 8, 10, 15, 20 nm).

For the construction of a multilayer assembly with SiNPs and cytochrome *c *the binding behavior of the modified SiNPs to a cyt *c *layer is important. This process has been studied with a cyt *c *monolayer adsorbed onto a mercaptoundecanol/mercaptoundecanoic acid (MU/MUA) modified gold chip using the QCM technique. As shown in Figure 5 a well-defined binding behavior of the 5 nm SiNPs at pH 7 at low ionic strength can be found. During the washing steps with buffer the signal for adsorbed SiNPs changes only slightly. Thus it can be stated, that only a small amount of the material is loosely bound. We have also investigated whether cyt *c *binds to the SiNP-layer. For an electrostatically driven process it can be expected that this adsorption is rather fast and almost comparable to the binding of cyt *c *to other negatively charged layers, such as SAMs or polyelectrolytes. By analyzing the binding behavior, it is obvious that there is a rather high amount of adsorbed cyt *c*, but not all protein molecules are tightly bound and a part of the cyt *c *can be washed away in the following washing step.

### Multilayer assembly of SiNPs and cyt *c*

On the basis of the binding experiments the construction of SiNP/cyt *c *-multilayers has been performed first on QCM gold chips to study the impact of the particle size on the binding performance by the use of different-sized SiNPs (5, 8, 10, 15, 20, 40, 60 nm, Figure 5). If the particle size exceeds 20 nm the binding and accordingly the formation of the multilayer assembly (cyt *c*/SiNP) can hardly be detected. Nevertheless, a recharging at the surface can be concluded since a further adsorption of SiNPs after the cyt *c *incubation is feasible. This may be attributable to a partial displacement of the larger SiNPs (20 and 40 nm) during the adsorption of cyt *c*. For the smaller sized SiNPs (Ø = 5 - 15 nm) however, successful assembly formations have been detected (Figure 5). The adsorbed amount of cyt *c *increases rather linearly with growing number of layers. For comparison polyelectrolyte-based protein assemblies with polyaniline sulfonic acid or DNA have shown an exponential layer growth. The reason for this difference can be attributed to a different structure of SiNP/cyt *c *-multilayers. The SiNP-layers seem to be more ordered than polyelectrolyte-based layers, because of a rather high homogeneity of the SiNPs and the absence of layer interpenetration which is discussed as one reason for exponential layer growth [39,40].

### Electrochemical characteristics of the multilayer assemblies (SiNPs/cyt *c*)

**Figure 6:**
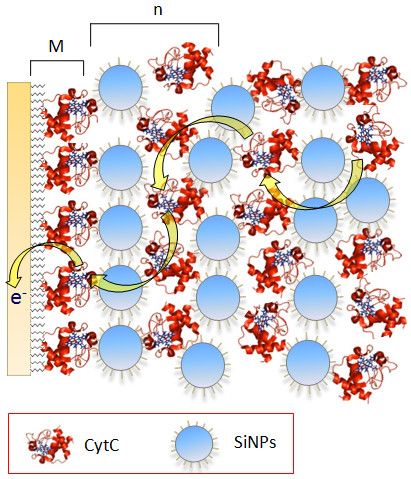
**Schematic representation of the SiNPs/cyt *c *multilayer architecture**. Schematic representation of an SiNPs/cyt *c *multilayer assembly prepared on a monolayer electrode (**M**). Layer structure [SiNPs/cyt *c*]_n _(**n **= 1, 2, 3, 4). Yellow arrows indicate the direct electron transfer between the cyt *c *molecules and onto the electrode.

The demonstrated binding forces between SiNPs and cyt *c *are strong enough for the arrangement of layered architectures, but this is only one precondition for a functional system with an electro-active protein. To ascertain the electrochemical characteristics, SiNP/cyt *c *multilayers have been prepared on a MU/MUA modified gold electrode. The SiNPs are introduced in the cyt *c *assemblies as schematically illustrated in Figure 6.

**Figure 7:**
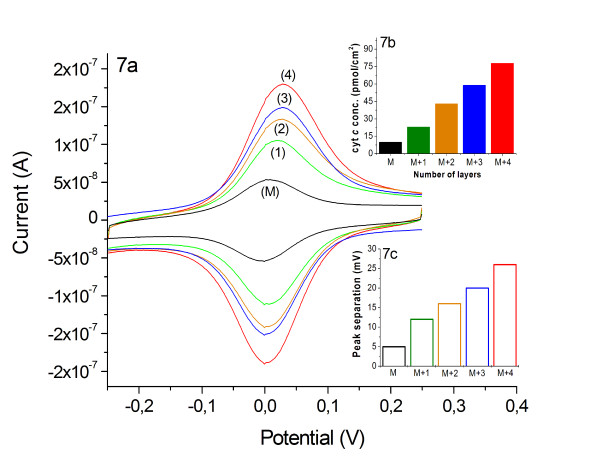
**Cyclic voltammetric integration of different multilayer assemblies**. **7a**) Cyclic voltammetry of 1, 2, 3, 4-bilayer assemblies of cyt *c*/SiNPs (Ø = 5 nm) and a cyt *c *monolayer (M) Au-MU/MUA-cyt *c *for comparison. Inset: **7b**) Electrochemical determined concentration of cytochrome *c *in the multilayer assemblies [1, 2, 3, 4-bilayers SiNPs (Ø = 5 nm)/cyt *c*], compared to a monolayer (M). Inset: **7c**) Peak separation of cyt *c *multilayer assemblies determined by cyclic voltammetry for 1, 2, 3, 4-bilayers of SiNPs/cyt *c *assembled on a monolayer (M). For the data represent in this figure a scan rate of 100 mV/s, KPP pH 7 was used.

Electrochemical studies using cyclic voltammetry show that cyt *c *in the protein-SiNPs assembly is electro-active. The electrode-addressable amount of cyt *c *molecules corresponds to an increase in cyt *c *loading on the electrode. Obviously the presence of SiNPs does not disturb the electron transfer through the assembly and cyt *c *can be addressed by the electrode at least up to 5 layers (Figure 7a). No direct electron transfer into the inert nanoparticles has been found in the potential range of -350 to +350 mV vs. Ag/AgCl. With raising number of layers the amount of electrochemically detectable cyt *c *increases. About 10 times more cyt *c *(80 ± 10 pmol/cm^2^) is found for a 5-layer assembly (cyt *c*/SiNP) than in a monolayer (Figure 7a). Control experiments in which either the SiNPs or the cyt *c *solution is replaced by buffer solution show only the response of a monolayer electrode, which demonstrates that both components are necessary for a successful formation of the multilayered protein assembly. The distinct increase of electro-active cyt *c *with growing number of deposited layers proofs the efficient electron transfer through the system (Figure 7b). If we take a closer look on the peak width at half peak height we can see, that the half peak width of the monolayer (130 ± 5 mV) and the multilayer assembly (132 ± 6 mV) are almost the same. This is indicative that cyt *c *molecules within the assembly are present in a rather similar state of heterogeneity (since the peak width is larger than 90 mV for a homogeneous state). So the introduction of SiNPs does not result in new states of the redox protein on the electrode surface. The peak separations found for SiNPs-based multilayer electrodes (5-layers: 26 ± 2 mV) is increased compared to that of a monolayer (5 ± 3 mV) but the absolute values are rather small (Figure 7c). This observation is an indication for a rather fast electron transfer between the cyt *c *molecules in the multilayer assembly.

An electron transfer rate constant k_s _for all cyt *c *molecules in the multilayer assembly cannot be given since the reaction rate decreases with increasing distance to the electrode. This circumstance can be seen by experiments with increasing scan rate; thereby a decrease in the electro-active cyt *c *amount is found. At higher scan rates cyt *c *molecules in the outer shells cannot take part in the redox conversion since the rate of potential change exceeds the rate of electron transfer through the assembly to the electrode. Therefore only a k_s_-range of 27-75 s^-1 ^can be given. Here the latter value reflects k_s _of cyt *c *immobilized in the first monolayer directly on the MU/MUA promoter, and the first value reflects k_s _of cyt *c *in a 5-layered cyt *c*/SiNPs (5 nm) assembly.

To gain a further impression on the reaction rate between the cyt *c *molecules within the assembly the self-exchange rate k_ex _for the immobilized cytochrome *c *is estimated. Therefore, the Dahms-Ruff equation has been used, assuming an intermolecular electron hopping process between the cyt *c *molecules in the multilayer film: $k_{ex}=\frac{6\;D_{effective}}{\delta^{2}\left[cyt\;c\right]}$

The effective diffusion coefficient D_effective _is calculated according to the Randles-Sevcik equation by evaluating the peak currents at small scan rates. For the cytochrome *c *(*horse heart*) in the multilayer, a D_effective _of 4.96 × 10^-12 ^cm^2 ^s^-1 ^is calculated. The approximate distance between the adjacent redox centers δ is estimated to be 2.6 nm and a reasonable cyt *c *concentration of 23 mM is used. With these values, a self-exchange rate constant of 1.99 × 10^4 ^M^-1 ^s^-1 ^for the cytochrome *c *in the multilayer is calculated. The literature values for k_ex _of horse heart cyt *c *in solution are in the range of 10^2 ^- 10^5 ^M^-1 ^s^-1 ^depending on the ionic strength. The rather high k_ex _for the cyt *c *in the multilayer assembly reflects the small values found for peak separation, and therefore is a further indication of a rather fast electron transfer within the cyt *c *multilayer system. In the SiNPs-based multilayer assemblies the formal potential is comparable to cyt *c *immobilized as a monolayer, e.g. on MU/MUA [41] and can be determined to be -24 ± 5 mV vs. Ag/AgCl.

In other multilayer systems using PASA or DNA as building blocks the electron transfer has been suggested to occur via protein-protein electron exchange [30,31]. But till this day no definite proof can be given that the polyelectrolyte or DNA are not acting as conducting mediators between the cyt *c *molecules within the assemblies. Based on the current work using non-conductive SiNPs it becomes clear that artificial multilayer architectures can be prepared with the interprotein electron exchange as the dominating mechanism.

**Figure 8:**
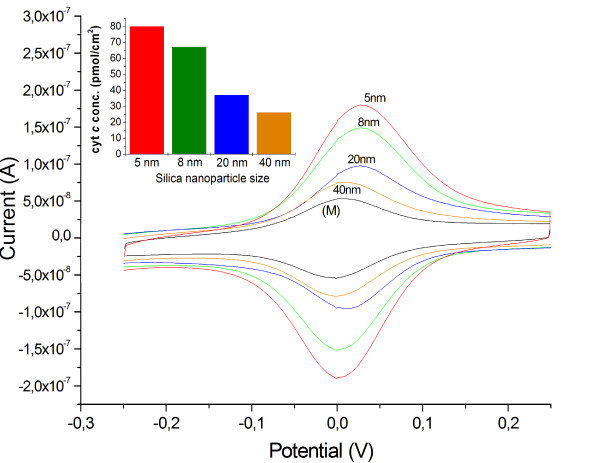
**CV - Multilayer with different-sized SiNPs**. Cyclic voltammetry of 4-bilayer-assemblies of cyt *c*/SiNPs: Ø = 5 nm, 8 nm, 20 nm, 40 nm and a cyt *c *monolayer (**M**) for evaluation of the influence of the SiNPs particle size (scan rate 100 mV/s, KPP7). Inset: Bar plot of the cyt *c *concentrations of 4-bilayer-assemblies (cyt *c*/SiNPs) with different-sized SiNPs.

To study the influence of the particle size on multilayer formation we perform series of experiments with larger SiNPs (5, 8, 20, 40 nm), see Figure 8. By cyclic voltammetric experiments for electrodes with the same number of layers (4-bilayer) a decrease in electro-active cyt *c *amount with increasing particle size (SiNPs) is found.

This observation reflects almost the results of the adsorption experiments by QCM. This means that the low electrochemical response for layer structures with larger particles is mainly caused by a less efficient layer formation process. The observations during this work anticipate that the nanoparticle size strongly influences cyt *c*-silica nanoparticle interactions.

In conclusion a certain balance on the interactive forces seems to be necessary for a stable layer formation on the one side and rotational flexibility of cyt *c *within the system for efficient electron exchange on the other side. This works best when both components of the assembly are of comparable size.

**Figure 9:**
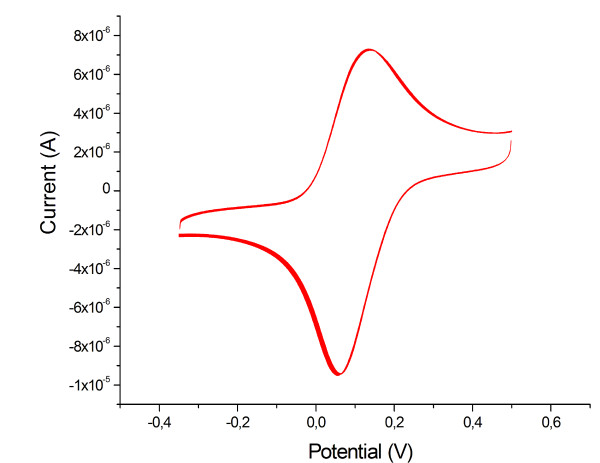
**CV - stability of the multilayer**. Multilayer stability on a QCM-chip of a 4-bilayer-assembly of cyt *c*/SiNPs (Ø = 5 nm) determined by cyclic voltammetry at a scan rate of 100 mV/s in KPP7 for 150 cycles.

In a further step of characterization the stability of the prepared multilayer architectures has been investigated. Figure 9 shows 150 voltammetric cycles for a 4-bilayer chip-electrode directly after the layer formation. The results illustrate a good stability of the assembled chip-electrode. It has to be pointed out that no thermal treatment is necessary as reported for polyelectrolyte-based systems and thus sufficient operational stabilities can be provided with the new strategy for arranging proteins on multiple electro-active layers.

## Conclusions

This study demonstrates the ability to construct fully electro-active cyt *c *multilayer assemblies by using carboxy-modified silica nanoparticles of small diameter (5 - 8 nm) as second building block. The absence of any conductive properties in the second building block clearly demonstrates that mechanisms for electron transfer through such protein multilayer assemblies is based on interprotein electron exchange, rather than on wiring of the protein to the electrode. Furthermore it is demonstrated that polymer-free cyt *c *multilayer assemblies can be built up.

Prepared on gold electrodes these multilayer architectures give a well defined, quasi-reversible response of cyt *c*. The formal potential of cyt *c *is not significantly influenced by the immobilization and is around -24 ± 5 mV vs. Ag/AgCl. However the peak separation is rather small. This is indicative for a fast and efficient electron transfer through the layered system. The peak width at half peak height for the multilayer system (132 ± 6 mV) is almost the same as for a cyt *c *monolayer (130 ± 5 mV). This shows that the cyt *c *molecules within the assembly are present in equal states, and thereby the protein molecules are expected to be bound in a rather similar microenvironment within the SiNPs matrix.

SiNP-based cyt *c *assemblies exhibit a linear increase of the cyt *c *amount with the number of adsorption steps as shown by the voltammetric response. For a 5-layer assembly an electro-active surface concentration of 80 ± 10 pmol/cm^2 ^can be found. A significant increase in the amount of protein with the number of layers is also verified by QCM. Furthermore it can be demonstrated that the cyt *c*/SiNP assemblies are stable without any thermal treatment at low ion strength at neutral pH.

The construction strategy of this multilayer system provides a new controllable route to immobilize proteins in multiple layers featuring direct electrochemistry without mediating shuttle molecules. Our system offers new possibilities particularly for biosensing. For this purpose, the use of a multilayer sensor with higher amounts of electro-active redox proteins (e.g. cyt *c*) can be expected to yield a significantly enhanced sensitivity.

The efficient reproducible DET and IET through several layers also provide an opportunity to simulate biological electron transport systems based on redox proteins. Hence the approach may be applicable to the construction of a new generation of signal chains for bioelectronic functionalities.

Further studies are thus directed to generalize the effects observed in this work to other proteins and biological molecules.

## Materials and methods

### Description of Chemicals

Mercaptoundecanoic acid (MUA), 11-mercapto-1-undecanol (MU), horse heart cytochrome *c *(cyt *c*), ammonium hydroxide (99.99%), tetraethyl orthosilicate (99.99%), ethanol absolute, (3-aminopropyl)-triethoxysilane, succinic acid anhydride, toluene anhydrous (99.8%) are purchased from Sigma-Aldrich (Steinheim, Germany), tetrahydrofuran spectranal is purchased from Riedel-de Haen (Seelze, Germany), di-potassium hydrogen phosphate, potassium di-hydrogen phosphate are provided by MERCK (Darmstadt, Germany), and gold-wire electrodes by Goodfellow (Bad Nauheim, Germany), Goldchips for QCM are delivered by QSense (Froelunda, Sweden). Two different buffers have been used during these investigations: 5 mM potassium phosphate buffer pH 7 and 1 mM potassium phosphate buffer pH 7.

### Synthesis of monodispersed SiNPs

Mono-dispersed spherical silica nanoparticles were synthesized following the Stöber-Fink-Bohn method starting from tetraethyl orthosilicate (TEOS), water, ammonia, and absolute ethanol, as precursor alkoxide, hydrolyzing agent, catalyst and solvent, respectively [3]. The overall experimental procedure is shown in Figure 1. Two mother solutions were prepared: one containing ammonia-water, the other containing TEOS-ethanol. The same volumes of the two solutions were always mixed in a thermostatically controlled water bath (45 ± 1°C). A micro feed pump Harvard Apparatus (Model 11 Plus) with a constant flow rate (5.0 mL/min) fed the starting solution A (TEOS, ethanol) into the reactor to solution B (ammonia, water, ethanol) at 45°C and vigorously stirring, thereafter the mixture prepared was agitated for 1 h to 5 d, dependent on the particle size to be synthesized. The SiO_2 _dispersion was transferred out of the reactor and centrifuged (at 4.000-14.000 rpm for 1 h). The precipitate was washed with ethanol by repeated centrifugation (at 4.000 -14.000 rpm for 1 h) and dried at 70°C for 12 h.

### Preparation of γ-aminopropyl modified silica nanoparticles SiO_2_-NH_2_

The amino groups modified SiO_2 _particles (SiO_2_-NH_2_), were prepared by the self-assembly of APTES onto the surfaces of SiO_2 _particles adapted from literature [42]. First 1.0 g of SiO_2 _particles was charged into a 25 mL three-necked round bottom flask containing 10 mL of dry toluene, and then the suspension was dispersed with ultrasonication for 30 min. Secondly the reaction flask was equipped with an N_2 _inlet, a thermometer, and a Graham condenser. Then, 2.5 mL of APTES was added quickly and the suspension was refluxed at 110°C for 12 h under N_2 _atmosphere and magnetic stirring. After the reaction was finished, the suspension was centrifuged at 4.500 - 14.000 rpm for 1 h and the precipitate was collected. Finally, the precipitate was redispersed into 25 mL of dry toluene with ultrasonication for 20 min and then centrifuged again. Next, the precipitate was dispersed into anhydrous ethanol with ultrasonication and centrifuged once again. The operation of dispersion and centrifugation was repeated for three cycles, and the resulting precipitate, SiO_2_-NH_2 _was dried under vacuum at 40°C for 24 h.

### Introduction of the carboxyl groups onto SiO_2_-NH_2 _particle surface

After the amino groups were grafted onto the SiO_2 _particle surface, they need to be coupled with succinc acid anhydride to introduce the carboxyl groups onto the surface. The coupling procedure was adapted from literature [43]. Briefly, SiO_2_-NH_2 _particles (1.0 g) were dissolved in anhydrous THF (50 mL) and then the suspension was dispersed with ultrasonication for 30 min. Then succinc acid anhydride (3.5 g) was added in two portions to the reaction suspension at 0°C and stirred for 2 h. Afterwards the reaction mixture was stirred at room temperature for another 24 h. The remaining succinc acid anhydride was hydrolyzed by addition of water. The resulting product (SiO_2_-COOH) was dispersed by ultrasonication for 15 minutes and centrifuged at 4,000 - 14,000 rpm for 1 h. Next, the precipitate was redispersed in anhydrous THF and centrifuged again. Finally, the precipitate was dispersed into water and centrifuged for another 30 min at 4,000 rpm, and the resulting precipitate, SiO_2_-COOH, was dried under vacuum at 40°C for 24 h.

### Characterization of the prepared silica particles

The synthesized SiNPs were characterized by ***dynamic light scattering analysis *(DLS)**. DLS was used to monitor the change in hydrodynamic radius (particle size) and aggregates. Measurements were carried out on a Beckman Coulter Delsa Nano C Particle Analyzer (Krefeld, Germany) working at a fixed angle of 90° in ethanol or water to obtain the number-average diameters of the particles. Each analysis was repeated three times to give the average particle size.

***Zeta-potential ***of the SiNPs was measured with a Beckman Coulter Delsa Nano C Zeta Potential Analyzer (Krefeld, Germany) and the measurement was repeated three times, and the average of them was reported as the final result.

***FT-IR analysis ***for monitoring the surface modification on SiNPs was measured by Fourier transform infrared spectroscopy (FT-IR) with a Varian 680-IR FT-IR spectrometer (Varian, Australia), ATR (Diamond), resolution 8 cm^-1^, scans 16, in the range of 400 - 4.000 cm^-1^.

***TEM measurements ***were applied to characterize the morphology and size of the different SiNPs, with a FEI Tecnai G^2 ^20 S-TWIN transmission electron microscope, 200 kV operation voltage, resolution 0.24 nm, EDAX EDX-system with a Si(Li)-detector, detection limit start at Bor (Z = 5).

### Fabrication of mono- and multilayer assemblies with SiO_2_-NPs

Gold-wire electrodes are cleaned by 2 times incubation in piranha solution (3:1 H_2_SO_4_/H_2_O_2_) for 10 min. The electrodes are washed with Millipore water after the cleaning steps. For the construction of multilayers the electrodes are modified by incubation for 48 h in 5 mM 3:1 solution of mercaptoundecanol/mercaptoundecanoic acid. The cyt *c *monolayers are prepared by incubation of the electrodes in 20 μM cyt *c *in KPP 5 mM pH 7 for 2 h [41]. The assembly of SiNPs/cyt *c *multilayers has been performed by alternating incubations of the cyt *c *monolayer electrode in 20 μM cyt *c *and SiNPs (0.3 - 5.0 mg/ml) for 10 min per step [31]. Each of the 10 minute long adsorption steps was followed by rinsing the electrodes in 5 mM KPP pH 7. The procedure was repeated until the desired number of layers was reached.

### QCM Measurements

A Q-Sense-D E4 piezoelectric instrument (QSense, Vaestra Froelunda, Sweden) was used for the quarz crystal microbalance measurements. A clean gold covered quarz sensor chip (5 MHz, QSense, Vaestra Froelunda, Sweden) was incubated in ethanol solution containing 5 mM MUA/MU (1:3) for 24 h, then rinsed with water and mounted into the QCM flow system. The solutions containing SiNPs and cyt *c *of above given concentrations were successively pumped through the cell for 10 minutes with 5 minutes of buffer flow in between, at a flow rate of 25 μL/min. We estimated the mass increase [*Δm *(ng)] from QCM frequency shift [Δ*f *(Hz)] of fixed films by using the Sauerbrey equation [44]. Taking into account the diameter of the electrode (d = 10 mm) and the other technical parameters [45], this equation can be written as: *Δm *= 1.03 *Δf*. According to this a layer mass increase can be estimated. Since measurements have been performed in solution, these values are estimated due to unknown amount of bound water.

### Electrochemistry

All electrochemical measurements were carried out in a 1 mL cell using an Ag/AgCl/1 M KCl reference (Microelectrodes, Inc., Bedford, USA) and Pt-wire counter electrode. The working electrodes were modified gold wires (diameter 0.5 mm) obtained from Goodfellow (Bad Nauheim, Germany) which are modified according to the procedures described above. Cyclic voltammetric experiments were carried out with CH Instruments CHI 660D device (Austin Texas, USA). Scan rates were varied between 0.01 and 50 V/s, but a scan rate of 100 mV/s was normally used. The potential range has been chosen between -0.3 and +0.3 V vs. Ag/AgCl. Data analysis has been performed using CHI 660D (Austin Texas, USA) software.

## List of abbreviations

**APTES**: (4-aminopropyl triethoxy silane); **CV**: (cyclic voltammetry); **cyt *c***: (cytochrome *c*); **D_effective_**: (effective diffusion coefficient); **DET: **(direct electron transfer); **DLS: **(dynamic light scattering); **DNA: **(desoxyribonucleic acid); **FT-IR: **(Fourier transformed infrared spectroscopy); **IET: **(interprotein electron transfer); **k_ex_: **(self-exchange rate constant); **KPP: **(potassium phosphate buffer); **MU: **(mercaptoundecanol); **k_s_: **(dissociation constant); **MUA: **(mercaptoundecanoic acid); **PASA: **(polyaniline sulfonic acid); **pH: **(***p****otentia ****h****ydrogenii*); **PI: **(polydispersity index); **QCM: **(quarz crystal microbalance); **SAM: **(self-assembling monolayer); **SiNPs: **(silica nanoparticles); **TEM: **(transmission electron microscopy); **TEOS: **(tetraethyl orthosilicate).

## Competing interests

The authors declare that they have no competing interests.

## Authors' contributions

**SCF **carried out all the experiments and prepared the manuscript. **FL **supervised the design of the study and the drafting of the manuscript. **All authors read and approved the final manuscript**.
